# EvoDesign: *de novo* protein design based on structural and evolutionary profiles

**DOI:** 10.1093/nar/gkt384

**Published:** 2013-05-11

**Authors:** Pralay Mitra, David Shultis, Yang Zhang

**Affiliations:** Department of Computational Medicine and Bioinformatics, University of Michigan, Ann Arbor, MI 48109 USA

## Abstract

Protein design aims to identify new protein sequences of desirable structure and biological function. Most current *de novo* protein design methods rely on physics-based force fields to search for low free-energy states following Anfinsen’s thermodynamic hypothesis. A major obstacle of such approaches is the inaccuracy of the force field design, which cannot accurately describe the atomic interactions or distinguish correct folds. We developed a new web server, EvoDesign, to design optimal protein sequences of given scaffolds along with multiple sequence and structure-based features to assess the foldability and goodness of the designs. EvoDesign uses an evolution-profile–based Monte Carlo search with the profiles constructed from homologous structure families in the Protein Data Bank. A set of local structure features, including secondary structure, torsion angle and solvation, are predicted by single-sequence neural-network training and used to smooth the sequence motif and accommodate the physicochemical packing. The EvoDesign algorithm has been extensively tested in large-scale protein design experiments, which demonstrate enhanced foldability and structural stability of designed sequences compared with the physics-based designing methods. The EvoDesign server is freely available at http://zhanglab.ccmb.med.umich.edu/EvoDesign.

## INTRODUCTION

The number of possible amino acid sequences is huge (∼20*^L^* with *L* being the sequence length). But only a few of them have folded into real proteins in nature that have a unique folding state with physiological activities. The driving force of such ‘nature protein design’ includes both physicochemical interaction and evolutionary pressure (1,2). Computer-based rational protein design aims to engineer novel sequences of stable folding states and in particular those with desirable physiological functionality. Technically, it can be considered as a reversal of protein folding that critically challenges our understanding of the fundamental principles of protein folding and stability (3–5). Protein design has also significant biomedical implications on its own. Successful protein designs and engineering have been shown to generate novel catalytic activities (6,7) and result in new therapeutic developments (8,9).

Most of the computer-based protein design efforts are based on Anfinsen’s thermodynamic hypothesis (10), which aim to identify new sequences of lowest free energy on various designed force fields. One obstacle in using physics-based approaches comes from the inaccuracy of the force field potentials for structural and thermodynamic optimization of the protein stability. Motivated by the superiority of template-based approaches in protein structure prediction, which construct structural models using evolutionarily related protein as template (11,12), we have developed an evolutionary profile-based method for *de novo* protein design (13), where sequence space search is constrained by the amino acid sequence profiles as computed from the homologous structure families. The physicochemical features of the designed sequence are smoothed by neural-network predictions of local structural features, including secondary structure, backbone torsion angle and solvation. The evolutionary profile-guided simulation search has the advantage to allow for designing and engineering proteins of larger size and more complex topology compared with that on physical force fields.

Here, we describe EvoDesign, an evolutionary profile-based web server for *de novo* protein design, which is developed based on our recent protein design method (13). The server offers several options for users to select different guiding force fields, structural thresholds for profile construction and residue conservations. The execution time of the server is fast and scales in hours because of the quick convergence of the simulation search under the profile restraints. EvoDesign is established as an automated, and yet reliable, on-line facility most useful for protein engineering and drug discovery studies.

## MATERIALS AND METHODS

**Figure 1.**
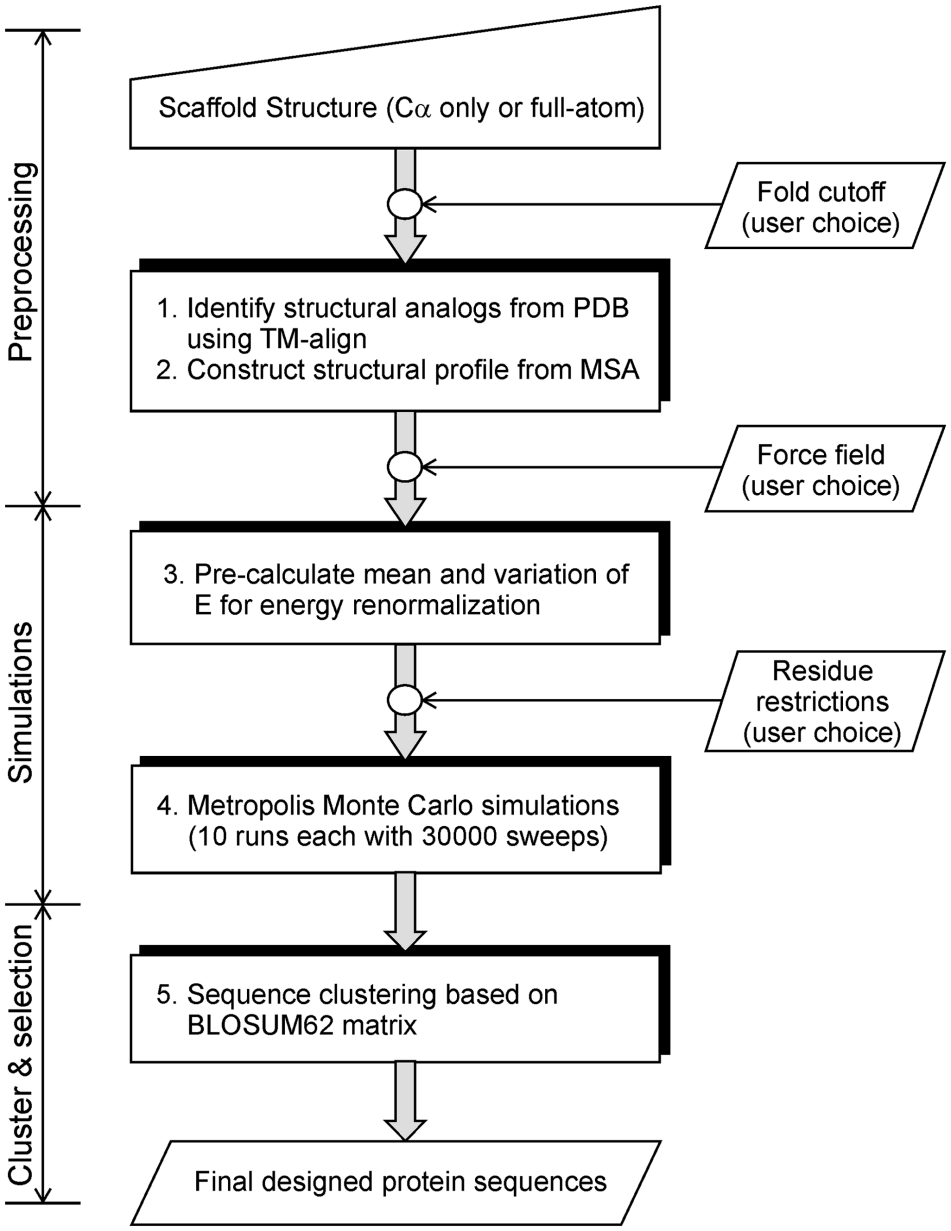
The overview of the EvoDesign server. The process is divided into three steps. Pre-processing and clustering take place in a single processor, whereas simulations are completed in parallel on 10 processors.

Figure 1 depicts a flow chart of the EvoDesign server, which is divided into three stages: (i) pre-processing: generation of scaffold-specific evolutionary profile restraints; (ii) simulation: Monte Carlo search on the sequence space; and (iii) clustering and selection: sequence clustering for design selection.

### Pre-processing

Starting from a scaffold protein structure, EvoDesign first collects a set of proteins of similar folds from the PDB library by the structural alignment program TM-align (14). By default, a high-structural similarity (TM-score >0.7) is used, which will be gradually reduced till the number of structural homologies is >10 or the TM-score threshold is equal to 0.5. Based on the preference in structural variations, users can control the diversity of the protein by specifying different lower-limit of fold cut-offs. An evolutionary profile is then constructed from the multiple sequence alignments that are constructed based on TM-align alignments. This profile will be used to guide the conformational search of amino acid sequence space in the next step of Monte Carlo simulation, where the physicochemical packing of side-chain and backbone atoms is accommodated by neural-network–based solvation, torsion angle and secondary structure predictions (13).

#### Force field design

The EvoDesign force field is a linear combination of four terms: (i) log-odds match between decoy sequence and the structure profile of the target scaffold; (ii) secondary structure (SS) match between decoy and target scaffold; (iii) backbone torsion angle (TA) match between decoy and target; (iv) match of solvent accessibility (SA) of residues between decoy and target. If the target structure from user input is full-atomic, the SS, TA and SA on target are pre-assigned by DSSP program (15). If the scaffold is C-α only, an atomic model including backbone and side-chain heavy atoms is quickly constructed using the statistical parameters collected from the PDB (16), which is then fed into DSSP to assign the structural features.

The SS, TA and SA value of decoy sequences is predicted from neural-network learning, which was mostly trained on the PSI-BLAST position-specific scoring matrix (PSSM) (17). As new decoys are generated at each step of movement, we trained the features separately on single-sequences, which is much faster than the PSSM predictors (∼5 min versus <1 s) but with comparable prediction accuracy.

As an option, the EvoDesign server also allows users to select a physics-based potential, which will be linearly combined with the evolution-based energy terms. The FoldX (version 3.0b5) is exploited to count for the physics-based energy terms, including hydrogen-bonding, electrostatics, van der Waals, steric, solvation and entropy interactions (18). As FoldX requires 3D structure for energy calculation, the backbone structure of decoys is first obtained by projecting the target scaffold to the decoy sequence using Needleman–Wunsch dynamic programming based on the evolutionary scoring terms. The side-chain conformation is then calculated by SCWRL V4.0 (19) before FoldX calculation.

### Monte Carlo simulation

The sequence space is searched by Metropolis Monte Carlo simulation. Following the idea of negative design where the bias is introduced against misfolded states (4,20), our Monte Carlo simulation is guided by the *Z*-score of the decoy energy
(1)
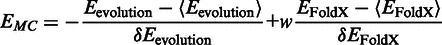

where 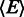 and δ*E* are average and standard deviation of energy scores calculated from baseline scores of 1000 random protein sequences. *w* = −2.44 or 0 when users select using or without using the physics-based force fields, respectively.

The simulation temperature is selected as 0.03. Ten simulation trajectories, each starting from different random sequences and running 30 000 sweeps, are conducted for a given target. If the job is submitted with evolution-based force field, the simulations are more than five times faster (but only slightly less accurate, see later in the text) than that using both evolution- and physics-based force fields.

### Sequence clustering and selection

In all, 290 000 decoy sequences from 10 different trajectories are pooled from the simulation, excluding the first 1000 sequences in each trajectory that are close to random. The sequence of the lowest free energy is selected by the SPICKER clustering algorithm (21) with the distance matrix between sequence decoys defined by BLOSUM62 substitution scores. Following the procedure by Bazzoli *et al.* (22), the distance threshold is initialized to zero, and then allowed to expand until the 40% of the sequences are included in the primary cluster. Top 10 seed sequences corresponding to the 10 largest clusters are output as the design sequences.

The method has been tested on 87 non-redundant proteins covering different fold classes (13). The data analysis showed that the evolution-based design significantly improves the foldability and ligand-binding affinity of the designed sequences compared with the traditional physics-based methods based on the computational validation of the designs (13,22). Without using homologous proteins, the designed sequences can be folded by the I-TASSER structural assembly simulation (23) with an average root-mean-square deviation (RMSD) 2.1 Å to the target. We have also used the method to redesign two cancer-related proteins, the X-linked inhibitor of apoptosis protein (XIAP) and the mouse double minute 2 homolog (MDM2), with the 3D structure and the peptide-binding affinity experimentally validated through circular dichroism, nuclear magnetic resonance spectroscopy and isothermal calorimetry experiments (D. Shultis, P. Mitra and Y. Zhang, submitted for publication).

## SERVER SETTING: INPUT, OUTPUT AND USER INTERFACE

### Input

The only input to the EvoDesign server is a scaffold structure of interest in PDB format. The minimal atoms needed in the file are C-α atoms, although including full-atom details may increase the accuracy of the structural feature assignments. User can customize their design by specifying: (i) fold similarity cut-offs (TM-score); (ii) inclusion of physics-based energy terms; and (iii) freezing specific residues by residue name or by residue number. By default, EvoDesign starts with a high TM-score threshold (TM-score >0.7) to construct profiles and exploits the evolution-based force field for free sequence design without any restrictions on residues.

The user has the option to control the fold-level homology for the construction of the structural profile. A higher homology (TM-score >0.7) insures a more accurate match with the scaffold (usually with a higher sequence identity as well), whereas a lower-homology threshold is an indication of incorporation of larger structure/sequence variability in the design. Generally, proteins with TM-score <0.5 to the scaffold do not retain the target structural fold well enough and are, therefore, excluded from profile construction in the default simulation. The evolution-based energy function alone is sufficient to design reasonable protein sequences based on our benchmark results. Moreover, it is faster than the combination of evolution and physics-based energy functions. Therefore, the default energy function for the EvoDesign server is set as evolution-based only. Option is also provided for users to exclude certain amino acids from the design at certain position, and/or to specify a set of residues (by residue number), which should be kept the same as in the input structure. This is particularly useful if the user has previous knowledge on the required function of the protein and does not want to (or prefer to) replace conserved residues, such as those that involved in ligand binding. The server needs a scaffold of at least 30 residues to achieve a meaningful fold.

### Output

**Figure 2.**
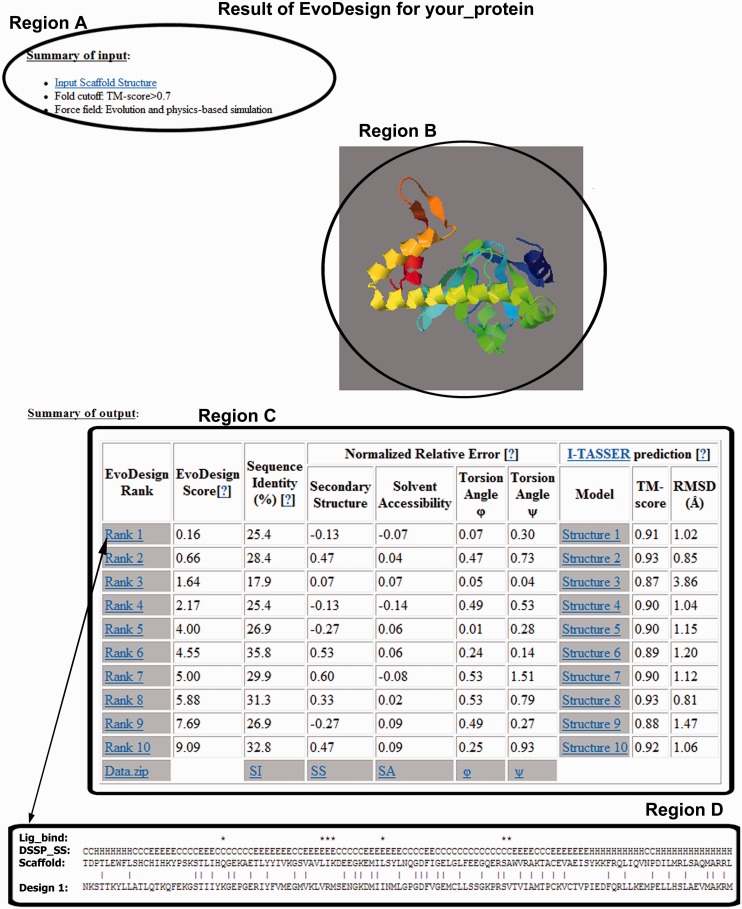
The screenshot of the result page of EvoDesign. The user choices and input structure are shown in region A and region B, which appears as soon as the job is submitted. The summary of EvoDesign will appear in region C after successful completion of the job.

The EvoDesign server provides users the design results and all assessment parameters of the target, with an illustration shown in Figure 2, which is taken from a snapshot of the output webpage sent to the users after the job is complete. A typical example of output is also available at http://zhanglab.ccmb.med.umich.edu/EvoDesign/example/index.php.

Region A and region B in Figure 2 appears in the output page as soon as the job is submitted. The original template scaffold structure is linked to the PDB format structure that was uploaded for design. If the uploaded structure is C-α only model, this will be linked to the full-atom structure file that is used for the protein design on successful completion of the job. The force field used for the design is also shown in this region. The image of the target scaffold structure is displayed through Jmol software [Jmol: an open-source Java viewer for chemical structures in 3D (http://www.jmol.org/)]. To view and operate on the image, the user needs to update the browser with current version of Java.

The summary of the design results is tabulated in region C that appears on the output page as soon as the design simulation is completed. EvoDesign outputs a maximum of 10 design sequences as listed in a decreasing order of the cluster size (or an increasing order of free energy), although the first-rank sequence is always suggested. The first column in the result table indicates the EvoDesign rank, followed by the EvoDesign score, which is the total confidence score of the designed sequences. The lower the EvoDesign score is, the higher the confidence is. In general, a design with the EvoDesign score <1.0 indicates a sufficiently high-confidence design, which corresponds to the model predictions with an average TM-score >0.7 and RMSD <2.0 Å in our benchmark test. The percentage of the sequence identity between the designed and the scaffold sequences is denoted at the third column.

Columns 4–7 present the estimated quality of the design sequence in term of normalized relative error (*NRE*) based on neural-network predictions. Here, the normalized relative error is defined as *NRE* = (*EDS* − *ETS*)/*ETS*, where *EDS* is the error of the neural-network predictions relative to the scaffold structure on the design sequence and *ETS* is the error of the predictions based on the sequence of the target scaffold. The secondary structure, solvent accessibility and backbone torsion angles are assigned using DSSP program for the scaffold structure (15). The predictions of secondary structure for target and design sequences are generated by PSSPred (24), an accurate SS predictor (with an average Q3 accuracy = 0.84 in 2000 test proteins) that combines seven neural-network predictors from different PSI-BLAST profiles and parameters. The torsion angles and solvent accessibility predictions of the sequences are generated by ANGLOR (25) and Chen and Zhou (26), respectively. Therefore, a negative *NRE* value indicates the design sequence has less prediction error relative to the scaffold structure compared with the scaffold sequence. Further details on the explanations can be found at the help page of the EvoDegin server (http://zhanglab.ccmb.med.umich.edu/EvoDesign/help.html).

To evaluate the foldability of the design sequences, columns 8–10 in the result table provide links to the structure modeling of the design sequences by I-TASSER and its structural deviation from the input scaffold. User can download the model structure in the PDB format from the links provided at the eighth column of the table. Last two columns present the TM-score and RMSD of the I-TASSER models on the designed sequence from the input scaffold. It should be noted that the I-TASSER structure prediction takes a few more hours on top of the EvoDesign. Thus, the output page first displays the design sequences along with all the sequence features (columns 1–7) as soon as the EvoDesign portion is completed. The I-TASSER results will be appended to the table when they become available.

The EvoDesign rank (first) column of the table links with a text file containing information of the design sequences. In this text file, the first row shows the ligand-binding positions (defined as residues within an 8 Å sphere radius of the hetero atoms) if the target scaffold structure contains hetero atoms. This facilitates a quick identification of the residue conservation at the hetero atom-binding sites. The second row is the DSSP secondary structure assignment with C/H/E indicating coil, helix and extended stand, respectively. The third and fifth row shows the scaffold and design sequences, respectively, where the identical residues between them are marked by ‘|’ at the penultimate row.

The bottom row of the result table is hyperlinked with the summary text file of sequence identity (SI), secondary structure prediction/assignment (SS), solvent accessibility (SA) and torsion angles (φ/ψ) of all 10 design sequences. User can download all of the information as a single zipped file (Data.zip: last row, first column).

Below the result table, a section ‘Detail on Design’ is added to present the detail information of each designed sequence, including the 3D models, sequence identity, secondary structure and other sequence-related features.

## ACCURACY VERSUS EFFICIENCY

The overall computing time of the EvoDesign server depends on the length of the scaffold and the force field selected for simulation. To test the impact of the force field selections on the accuracy and efficiency of the EvoDesign server, we arbitrarily selected seven non-homologous proteins with varied length and different SCOP class (27). Sequences of the selected scaffolds are designed using the EvoDesign server without and with physics-based force field.

As shown in the columns 4–7 in Table 1, the Monte Carlo simulation and pre-processing steps take the majority of the running time of EvoDesign, where the computing time of the sequence clustering stage, which uses a highly optimized SPICKER algorithm (21), is almost negligible. Overall, the EvoDesign server with physics-based force field takes 5.2-fold longer time than that without the physics-based force field. A detailed investigation on the simulations indicates that the increase in time is mainly because of the computationally intensive SCWRL program (19), which takes up to several minutes to construct side-chain conformations on a single sequence.

**Table 1.** Accuracy versus efficiency of the EvoDesign server on seven non-homologous proteins at different sizes

			Computational time (h)				Goodness of design							
			Pre-processing	Simulation	Clustering	Total	Sequence identity (%)	Normalized relative error (NRE)				RMSD (Å)^b^		
DB ID (SCOP class^a^)	Protein length	Included physics-based force field?						Secondary structure	Solvent accessibility	Torsional angles		I-TASSER	SPARKS-X	Rosetta
										Φ	Ψ			
1GUT_A (b)	52	No	0.5	3.8	0.1	4.4	22	1.0	−0.2	−0.0	−0.1	0.8	1.2	2.9
		Yes	1.6	16.5	0.1	18.2	31	0.5	0.0	−0.1	−0.3	0.5	1.1	5.2
1V5I_B (a + b)	71	No	0.7	4.4	0.1	5.2	11	0.0	0.2	0.4	1.1	3.3	3.4	9.5
		Yes	2.5	16.3	0.1	18.9	24	−0.2	0.2	0.4	1.3	1.5	4.7	8.2
1BKR_A (a)	109	No	1.0	5.8	0.2	7.0	27	0.1	0.0	0.1	−0.1	0.4	1.9	5.3
		Yes	4.6	24.1	0.2	28.9	30	0.2	−0.0	0.1	−0.1	0.3	1.2	2.7
1T3Y_A (a + b)	132	No	1.3	6.7	0.2	8.2	18	−0.0	0.1	0.0	−0.1	1.9	2.0	4.9
		Yes	6.5	59.0	0.2	65.7	24	−0.2	−0.0	0.2	−0.2	1.8	2.4	5.5
2GMY_A (a)	148	No	1.5	7.4	0.3	9.2	14	1.0	0.0	0.4	0.3	1.0	1.8	12.4
		Yes	5.0	34.0	0.3	39.3	20	0.4	0.0	0.3	0.2	0.3	3.1	9.9
1Y25_A (a/b)	165	No	1.5	8.1	0.3	9.9	17	0.2	0.3	0.3	0.4	2.3	6.7	10.8
		Yes	6.6	58.3	0.3	65.2	29	0.1	0.1	0.2	0.1	1.2	1.6	14.0
2PTH_A (a/b)	194	No	1.9	9.0	0.3	11.2	12	0.7	0.6	0.7	0.8	1.7	17.1	16.7
		Yes	7.3	45.5	0.3	53.1	20	0.7	0.3	0.4	0.6	0.9	2.1	15.4
Average		No	1.2	6.4	0.2	7.9	17	0.4	0.1	0.3	0.3	1.6	**4.9**	**8.9**
		Yes	4.9	36.2	0.2	41.3	25	0.2	0.1	0.2	0.2	0.9	**2.3**	**8.7**

^a^(a) means class-α and (b) means class-β.

^b^RMSD is computed between the target scaffold and the model structure by I-TASSER, Rosetta and SPARKS-X on the design sequence (see Supplementary Table S1 for detail results, including TM-score and alignment coverage in both first and the best in top 10 models).

As a reward, the inclusion of the physics-based force field slightly decreases the *NRE* of SS, SA and TA and increases the sequence identity between design and target sequences (see columns 8–12 of Table 1). When we apply the I-TASSER program to fold the design sequences (where homologous templates with a sequence identity >30% to the target are excluded), all the design sequences can be folded to a model of correct fold, with the average RMSD = 0.9 and 1.6, for the sequences with and without combining the physics-based force field, respectively, which also indicates an improvement by FoldX. These data demonstrate that the evolution-based force field is sufficient to complete medium- to high-resolution sequence design (with RMSD varying from 0.4 to 3.3 Å) on its own. The inclusion of physics-based force field can help slightly improve the accuracy of local structural packing but significantly increases the server response time. Nevertheless, we recommend the inclusion of physics-based force field if user is interested to do more detailed study on the design sequence.

As further validations of the foldability, we submit the designed sequences to two independent programs Rosetta (28) and SPARKS-X (29), which represent two typical approaches of *ab initio* fold and fold recognitions. As expected, as Rosetta does not use global templates, it is only able to fold small proteins. If we count the proteins with a length <132 residues, Rosetta can generate correct fold with a TM-score >0.5 for all four targets when considering the best in top 10 predictions (Supplementary Table S1 in Supplementary Material). The average RMSDs are 3.9 and 4.1 Å for the EvoDesign sequences designed with and without using physics-based potentials, respectively. If counting the first model, however, the average RMSDs for the four proteins increase to 5.4 and 5.7 Å, respectively (Table 1), which are generally consistent with the performance of the *ab initio* structure modeling on the natural proteins (28).

Starting from the EvoDesign sequences, SPARKS-X can identify correct template with a RMSD <5 Å or TM-score >0.5 as the first model for all but one (PDB ID: 2PTHA) proteins (Table 1 and Supplementary Table S1). If we consider the best in top 10 templates, all the sequences have a correct template identified by SPARKS-X with TM-score >0.5 (Supplementary Table S1). The sequences designed with physics-based force field have a slightly lower RMSD than that without the force field (0.9 versus 1.5 Å for the best in the top 10, or 2.3 versus 4.9 Å for the first model, with the average alignment coverage >90% in all cases).

The front end of the EvoDesign server is designed in PHP (version 5.3.3) and HTML, whereas the back end is implemented using C, C++, Perl and FORTRAN. The low-level features of C and C++ help to optimize the code wherever possible. The back-end computations are conducted in a Linux cluster of 300 HP DL1000h octa-core nodes.

## CONCLUSION

We developed a new EvoDesign web server for *de novo* protein design, which identifies new protein sequences based on an evolutionary profile-guided Monte Carlo simulation search. The physicochemical packing of local structures is accommodated by single-chain–based neural-network training on secondary structure, torsion angle and solvent accessibility. An optional physics-based force field can be added to further improve the structural packing characteristics.

EvoDesign takes the structural coordinates of the scaffold protein as the only input and outputs the designed sequences along with the detailed quality analyses. The quality estimations of design sequences are particularly important, as it provides users with a comparison study with the target scaffold in terms of sequence identity, normalized relative error on secondary structure, solvent accessibility and backbone torsional angles, along with the conservation of hetero atoms-binding sites analyses. Meanwhile, a combined EvoDesign score is provided to assess the overall confidence of the designed sequences. The server also generates structural models from the state-of-the-art protein structure prediction algorithms, where the TM-score and RMSD of the predicted model to the input scaffold will provide additional assessment of the design confidence (Figure 2).

To facilitate specific requirements, the server provides options for users to select different force field combinations, structural fold cut-offs and conserved residue regions of simulation searches. As EvoDesign needs to construct the evolutionary profiles from similar protein folds in the PDB, it is critical for EvoDesign to have a complete and updated template library. Currently, a representative PDB structural library is maintained and updated weekly for EvoDesign at http://zhanglab.ccmb.med.umich.edu/library. Meanwhile, a message board is set-up at http://zhanglab.ccmb.med.umich.edu/bbs/?q=forum/2 to allow users to report feedback and discuss problems with authors.

It should be mentioned that many methods in the literature were developed to design proteins with either improved functions or completely novel folds. One motivation for the EvoDesign algorithm is to provide a reliable platform that can design any proteins with improved fold stability using the restraints from evolutionary profiles of similar fold families. With this platform, the functional characteristics, including the enhanced and alternative ligand bindings for instance, can be further introduced. In a recent achievement (P. Mitra, D. Shultis and Y. Zhang, in preparation), we have demonstrated that the introduction of specific interface potentials can drastically improve the binding affinity of natural or drug ligands on the designed proteins. We plan to integrate the ligand-binding potentials, together with other biologically function-oriented developments, to the EvoDesign server in near future.

Last but not the least, as the EvoDesign relies on the profile collections from solved structures in the PDB, it can raise the issue that the method may hamper the possibility in designing proteins of novel folds (5). With the rapid increase of the solved protein structures, however, the PDB library has approached to its completeness. As demonstrated by the recent studies (30–32), all single-domain protein structures, including the artificial polyalanine-chain models made by the computer-based assembly requiring only hydrogen-bonding and compactness, can find analogous proteins of similar fold in the PDB using the state of the art structural alignment algorithms. In this sense, there are essentially no (or very rare) new folds outside the PDB library; the current method should be in principle used to design sequences for any protein scaffolds considering the increasing completeness of the PDB library.

## SUPPLEMENTARY DATA

Supplementary Data are available at NAR Online: Supplementary Table 1.

## FUNDING

Funding for open access charge: National Science Foundation Career Award [DBI 0746198]; National Institute of General Medical Sciences [GM083107 and GM084222].

*Conflict of interest statement.* None declared.
